# Mycosis Fungoides of the Oral Cavity: Fungating Tumor Successfully Treated with Electron Beam Radiation and Maintenance Bexarotene

**DOI:** 10.1155/2016/5857935

**Published:** 2016-12-15

**Authors:** Juri Bassuner, Roberto N. Miranda, Drew A. Emge, Beau A. DiCicco, Daniel J. Lewis, Madeleine Duvic

**Affiliations:** ^1^The University of Texas MD Anderson Cancer Center, Houston, TX, USA; ^2^Baylor College of Medicine, Houston, TX, USA; ^3^University of Texas Medical School at Houston, Houston, TX, USA

## Abstract

Oral involvement in mycosis fungoides is unusual and portends a poor prognosis. The clinical findings of three new cases are described along with a differential diagnosis and review of the literature. For brevity, only one patient is discussed in detail below whereas the other two cases are solely described in table form. The patient had a four-year history of mycosis fungoides before developing an exophytic tongue tumor. He was treated with local electron beam radiation and is disease-free to date while being on maintenance therapy with oral bexarotene. Analysis of the data collected from our review of the literature and the present cases reveal key insights.

## 1. Introduction

One of the most common T-cell lymphomas is mycosis fungoides (MF). It is a malignant, insidious, cutaneous, extranodal non-Hodgkin's lymphoma (NHL) [1]. MF encompasses about 4% of all lymphoma cases worldwide and has an incidence of 0.36 per 100,00 [2]. The MF disease process has a relatively predictable pattern: in three phases, erythematous or eczematous patches can become infiltrated plaques and cutaneous tumors [1]. Extracutaneous manifestations of MF can involve a wide array of sites, particularly lymph nodes [3].

Oral cavity involvement in MF is rare, found in less than 1% of patients. Interestingly, autopsy studies suggest up to 13% involvement [4]. This is thought to be a predictor of poor prognosis. Often, patients have advanced stage disease and the majority have expired shortly after presentation [5–8]. We present a case of oral MF and a review of the literature. Two additional patients with oral MF that presented to our hospital are presented in table form alongside the 45 patients with oral MF found in the literature (Table 2). Key observations are made from analysis of the patients.

## 2. Materials and Methods

We have expanded on our previous case series on oral MF (20) to include three new cases that were selected from the electronic medical records of The University of Texas MD Anderson Cancer Center (UTMDACC). The patients were treated at UTMDACC over periods from 2005 to present (Case  1), 2005 to 2008 (Case  2), and 2015 to present (Case  3).

## 3. Case Report

A 63-year-old white man (Case  1) presented in 2005 with exfoliative erythroderma. He stated that he was diagnosed with a rash localized to his right hand three years earlier. Over the course of one and a half years, his lesions spread widely. On presentation, he had 90% body surface area (BSA) involvement with a 3 : 1 ratio of plaque to patch. His skin exhibited indurated erythematous papular rash that was confluent over the upper and lower extremities with skip areas on the abdomen and relative sparing of the groin.

Flow cytometry revealed 30 × 10^9^/L CD4 cells and 96% CD4+/CD26− cells. Biopsy of the tumor showed MF with large cell transformation.

The patient received numerous systemic treatments including (1) vorinostat 400 mg daily that improved his pruritus but was accompanied by intolerable side effect of diarrhea and overall lack of response in the skin, (2) forodesine with minor partial response, (3) combined modality with interferon-alpha plus bexarotene and extracorporeal photophoresis, (4) total body skin electron beam radiation that effectively cleared his skin temporarily, and (5) alemtuzumab with which he achieved durable near-complete remission.

**Figure 1:**
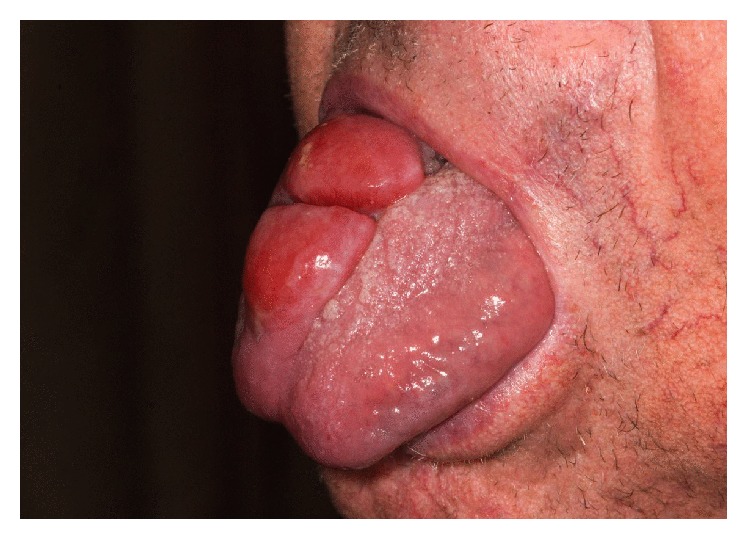
Mycosis fungoides tumor of the patient's tongue measuring 2.0 × 2.0 × 2.5 cm with a central cleft prior to treatment. The tumor was responsive to local electron beam radiation and maintenance bexarotene.

**Figure 2:**
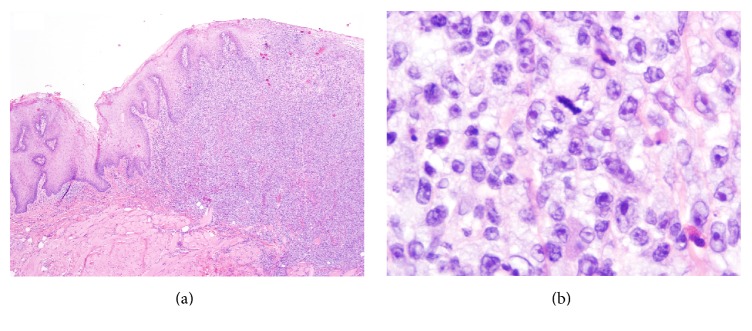
(a) Fungating lesion of the tongue shows a dense lymphoid infiltrate lined by the squamous epithelium of the oral mucosa. The infiltrate permeates into underlying skeletal muscle of tongue. Hematoxylin and eosin, ×40. (b) The infiltrate is composed of large cells with vesicular nuclei and prominent nucleoli. Atypical mitoses are also observed. Hematoxylin and eosin, ×1000.

**Figure 3:**
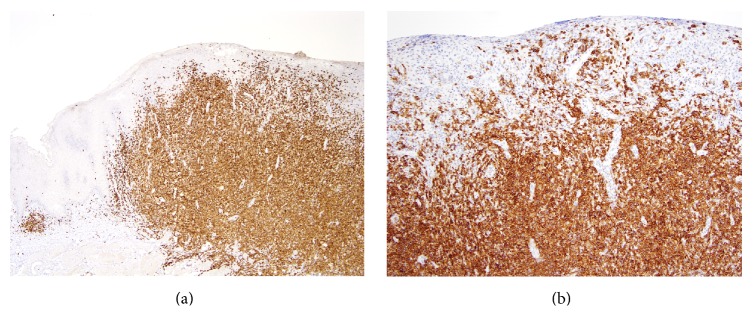
Immunohistochemistry shows that the large neoplastic cells are positive for CD3 (a) and CD30 (b). Immunohistochemistry with hematoxylin counterstain; ×40 (a) and ×100 (b).

After these treatments, roughly four years after initial presentation, the patient presented with a rapidly growing tumor on his tongue measuring 2.0 × 2.0 × 2.5 cm with a central cleft (Figure 1). His skin at that point had 12% BSA involvement of MF. His tongue biopsy showed a large protruding lesion, lined by squamous mucosa, nonulcerated, composed of a diffuse, dense lymphoid infiltrate that extended deep into underlying skeletal muscle of tongue (Figure 2(a)). On higher magnification, the neoplastic cells were large, with vesicular nuclei and prominent central nucleoli (Figure 2(b)). Approximately 2 atypical mitotic figures per high power field were identified. The large neoplastic cells were strongly and diffusely positive for CD3 (Figure 3(a)) CD4 and CD30 (Figure 3(b)). Approximately 90% of neoplastic cells expressed the proliferation marker Ki-67. Bone marrow was positive for atypical cells as well. Imaging revealed a 1.3 cm spiculated lesion in the left upper lobe, which was subsequently biopsied and found to be positive for lymphoma. His tumor responded to 22 Gy of electron beam radiation leaving behind a 3.0 × 1.5 cm erosion that eventually formed a scar. He was restarted on bexarotene and had an excellent response on the skin. He continues to be disease-free to the time of this writing.

## 4. Discussion

Lymphomatous lesions of the oropharynx in MF are becoming increasingly recognized in the literature. Presentation is heterogeneous, ranging from depressed ulcerations and red or white patches to exuberant outgrowth of tumors. This presents a diagnostic challenge to the uninitiated clinician. The differential diagnosis of various benign and malignant oral lesions is reviewed (Table 1).

MF is classically divided into three progressive, often overlapping, stages: patch, plaque, and tumor. Clinically and histopathologically, patch stage MF is commonly misdiagnosed as psoriasis. Lesions appear erythematous and sometimes scaly usually responding to topical steroids, the mainstay treatment [9]. Microscopically, there is nonspecific inflammatory infiltrate. Atypical cells are not readily identified.

During the plaque and tumor stages, lesions present a much more characteristic histologic picture. There are a dense polymorphous infiltrate and characteristic epidermotropism. Malignant cells called Sezary cells may be seen in the peripheral blood and subsequently may spread to lymph nodes. Sezary cells can be identified in peripheral blood by flow cytometry immunophenotype [10]. Treatments are often directed systemically with medicines such as bexarotene, a vitamin A derivative. In our experience, lesions respond well to local electron beam radiation.

To our knowledge, there are 42 reported cases of oral MF (Table 2). At presentation of oral MF, the age ranged from 36 to 81 years, with a median of 64. Forty percent were women and 60% were men. Skin involvement universally preceded oral involvement with the exception of two cases ranging from 6 months to 20 years, with a median of 4 years.

At time of oral lesion diagnosis, 33% of patients had stage IB disease or lower and 11% had no active cutaneous disease. Most commonly, patients presented with oral lesions on the palate (*n* = 21) and/or tongue (*n* = 20), which is consistent with the literature [8, 11–19]. Sixty-one percent had multiple sites of oral involvement. Of the lesions identified, there were 12 tumor, 11 plaque, and 3 patch.

Our patient is remarkable in that he is in complete remission seven years after onset of oral lesion, which defies the median time of one year from diagnosis of oral lesion to death. Further, our patient had large cell transformation, which carries additional poor prognosis [20]. At the time of oral lesion development, no lymphadenopathy was present whereas in many of the reported cases, oral lesions occurred mostly in advanced stages of the disease.

## Figures and Tables

**Table 1:** Differential diagnosis of oral tumors.

Disease	Oral lesion description	Diagnostic clues
Malignancy/premalignancy		
Squamous cell papilloma	Discrete exophytic papillary lesions (verruca): occur at any intraoral site	History of human immunodeficiency virus infection; association with cutaneous warts on fingers
Squamous cell carcinoma	Nonhealing ulcers, papules, or plaques: occur most frequently at the floor of the mouth and soft palate	History of tobacco and alcohol consumption; mechanical trauma from ill-fitting dentures

Mesenchymal neoplasms and tumor-like lesions		
Fibrous and vascular overgrowths	Discrete lesions of cheek or tongue	History of chronic irritation, usually from some tooth-related cause or chronic cheek/tongue biting
Pyogenic granuloma	Exuberant overgrowths usually at the gingiva but can occur at any intraoral site	May bleed spontaneously or following irritation due to extreme vascularity

Odontogenic tumors and cysts		
Ameloblastoma	Oral swellings occurring on the mandible that typically produce multicystic appearance on radiograph	Painless and slow growing; untreated, may reach substantial size
Odontogenic cysts	Oral swellings arising adjacent to teeth that usually produce a well-demarcated cyst on radiograph	Painless and slow growing

**Table 2:** Data from the literature where oral mycosis fungoides was identified before death. An asterisk denotes that the value was not stated or unknown.

Author	At onset of MF	At onset of oral lesion	At death	Time to death from onset of oral lesion (yr.)	Sex	Stage	Cutaneous involvement	Extracutaneous involvement	Lymph node involvement	Multiple sites of oral involvement	Presence of GI involvement	Lesion type	Location of oral lesion(s)
	Age					At onset of oral lesion							
Laskaris	52	65	65.2	0.2	F	IIb	+	+	+	+	*∗*	*∗*	Buccal mucosa, lips
Crane	70	73.5	*∗*	*∗*	F	IIa	−	−	−	−	−	*∗*	Gingiva
Yao	54	57.8	59.1	1.3	M	Ib	+	*∗*	*∗*	+	*∗*	Patch	Gingiva, buccal mucosa
Brousset	47	50	52	2	F	Ib	+	−	−	−	−	Tumor	Lingual margin
Vicente													
Case 1	51	59	59.5	0.5	F	IIb	+	−	−	+	*∗*	Plaque	Hard palate, mandibular gingiva
Case 2	72	77	77.5	0.5	F	IIb	+	*∗*	*∗*	−	*∗*	Plaque	Hard palate
Kasha													
Case 1	65	66	67.2	1.2	M	IIb	+	−	−	−	−	Plaque	Dorsal tongue
Case 2	62	80	81	1	M	IIa	+	−	+	+	+	Plaque	Tongue, esophagus
Evans	52	65	66.2	1.2	F	Ib	+	−	*∗*	+	*∗*	Plaque	Dorsal tongue, Lateral tongue
Barnett	39	69	69.2	0.2	M	IIb	+	*∗*	*∗*	+	*∗*	Plaque	Palate, tongue, mucosa, gingiva, pharynx
Cohn	50	52.5	*∗*	*∗*	M	IIb	+	+	+	+	*∗*	Plaque	Hard palate, buccal mucosa, tongue
Damm	68	68	*∗*	*∗*	M	IIb	+	−	−	+	−	*∗*	Hard palate, soft palate, alveolar ridge
Whitbeck	68	72	72.6	0.6	M	IVb	+	+	−	+	−	Tumor	Hard palate and, later, tongue
Ellams	52	52	52.3	0.3	F	Ib	−	−	*∗*	+	*∗*	Tumor	Gingiva, buccal mucosa, palate
Reynolds	60	75.5	76.7	1.2	F	Ib	+	−	*∗*	+	*∗*	Patch	Tongue, hard palate
Wright	60	61.5	62.7	1.2	M	IVb	+	+	−	+	*∗*	Patch	Hard palate, upper gingiva
Sirois													
Case 1	71	75	76	1	M	IVa	+	*∗*	*∗*	+	*∗*	*∗*	Gingiva, palate, tongue, lip, buccal mucosa, tonsil
Case 2	44	57	58	1	M	III	+	*∗*	*∗*	−	*∗*	*∗*	Tongue
Case 3	46	49	50	1	M	IVa	+	*∗*	*∗*	+	*∗*	*∗*	Gingiva, tongue
Case 4	71	74	75	1	M	IIb	+	*∗*	*∗*	+	*∗*	*∗*	Gingiva, palate
Case 5	62	66	69	3	F	IIb	+	*∗*	*∗*	+	*∗*	*∗*	Gingiva, palate
Case 6	51	53	56	3	F	IVa	+	*∗*	*∗*	−	*∗*	*∗*	Gingiva
Case 7	67	73	81	8	F	Ib	−	*∗*	*∗*	−	*∗*	*∗*	Gingiva
Case 8	43	51	53	2	M	III	+	*∗*	*∗*	−	*∗*	*∗*	Tongue
McBride	*∗*	63	63.1	0.1	F	IIa	+	*∗*	*∗*	−	*∗*	Tumor	Dorsal tongue
Harman	*∗*	57	57.6	0.6	M	IIb	+	−	−	+	−	*∗*	Gingiva, palate
Cawley													
Case 1	72	72	74	2	M	Ib	+	+	−	−	−	*∗*	Hard/soft palate, tonsils
Case 2	65	65	65.0	0.04	M	IIb	+	+	+	+	−	Tumor	Labial commissure, tongue
Postorino et al.	*∗*	60	*∗*	*∗*	M	IIb	+	−	+	−	−	Plaque	Mucosa
Corbett et al.	*∗*	*∗*	*∗*	*∗*	F	IIb	+	−	−	+	−	Tumor	Soft palate, throat
Wain et al.	*∗*	*∗*	*∗*	*∗*	M	Ib	+	−	−	+	−	Plaque	Soft palate, tongue, lips
Wahie et al.	60	69	*∗*	*∗*	M	Ia	−	−	−	+	−	*∗*	Suprahyoid region, epiglottis
Viswanathan	69	69	*∗*	*∗*	M	Ia	+	−	−	+	−	*∗*	Tongue, soft palate
Luigetti et al.	27	38	*∗*	*∗*	F	*∗*	+	+	+	+	−	Plaque	Lip, mucosa, tongue, pharynx
Goldsmith et al.	44	64	*∗*	*∗*	F	*∗*	+	−	−	−	−	Plaque	Hard palate
Le et al.	32	36	*∗*	*∗*	M	IIb	+	+	+	−	−	Tumor	Tonsil
Tillman et al.		60	*∗*	*∗*	M	*∗*	*∗*	*∗*	*∗*	*∗*	*∗*	*∗*	*∗*
Chua et al.	80	80.7	*∗*	*∗*	M	Ib	+	−	−	−	−	Tumor	Hard palate, gingiva, mucosa
Gomez													
Case 1	35	45	45.5	0.5	F	IIb	+	−	−	+	−	Tumor	Tongue, uvula, oropharynx
Case 2	66	70	*∗*	*∗*	F	Ib	+	−	−	+	−	*∗*	Uvula, soft palate, tonsils
May													
Case 1	*∗*	40	*∗*	*∗*	F	Ia	+	−	−	−	−	Tumor	Tongue
Case 2	44	44	*∗*	*∗*	M	*∗*	−	−	−	−	−	*∗*	Tongue
Present report													
Case 1	60	74	*∗*	*∗*	M	IVb	+	+	−	−	−	Tumor	Tongue
Case 2	50	55	55.7	0.7	M	IVb	+	+	−	+	−	Tumor	Palate, uvula
Case 3	35	38	*∗*	*∗*	M	IVb	+	+	*∗*	+	+	Ulcer	Tongue, palate

## References

[1] Ahn C. S., ALSayyah A., Sangüeza O. P. (2014). Mycosis fungoides: an updated review of clinicopathologic variants. *American Journal of Dermatopathology*.

[2] Criscione V. D., Weinstock M. A. (2007). Incidence of cutaneous T-cell lymphoma in the United States, 1973–2002. *Archives of Dermatology*.

[3] Burg G. (2015). Systemic involvement in mycosis fungoides. *Clinics in Dermatology*.

[4] Epstein E. H., Levin D. L., Croft J. D., Lutzner M. A. (1972). Mycosis fungoides: survival, prognostic features, response to therapy, and autopsy findings. *Medicine*.

[5] de la Fuente E. G., Rodriguez-Peralto J. L., Ortiz P. L., Barrientos N., Vanaclocha F., Iglesias L. (2000). Oral involvement in mycosis fungoides: report of two cases and a literature review. *Acta Dermato-Venereologica*.

[6] Jones D., Vega F., Sarris A. H., Medeiros L. J. (2002). CD4- CD8- ‘double-negative’ cutaneous T-cell lymphomas share common histologic features and an aggressive clinical course. *American Journal of Surgical Pathology*.

[7] Kasha E. E., Parker C. M. (1990). Oral manifestations of cutaneous T cell lymphoma. *International Journal of Dermatology*.

[8] Quarterman M. J., Lesher J. L., Davis L. S., Pantazis C. G., Mullins S. (1995). Rapidly progressive CD8-positive cutaneous T-cell lymphoma with tongue involvement. *The American Journal of Dermatopathology*.

[9] Wilcox R. A. (2016). Cutaneous T-cell lymphoma: 2016 update on diagnosis, risk-stratification, and management. *American Journal of Hematology*.

[10] Jawed S. I., Myskowski P. L., Horwitz S., Moskowitz A., Querfeld C. (2014). Primary cutaneous T-cell lymphoma (mycosis fungoides and Sezary syndrome): part I. Diagnosis: clinical and histopathologic features and new molecular and biologic markers. *Journal of the American Academy of Dermatology*.

[11] Damm D. D., White D. K., Cibull M. L., Drummond J. F., Cramer J. R. (1984). Mycosis fungoides: initial diagnosis via palatal biopsy with discussion of diagnostic advantages of plastic embedding. *Oral Surgery, Oral Medicine, Oral Pathology*.

[12] Evans G. E., Dalziel K. L. (1987). Mycosis fungoides with oral involvement. A case report and literature review. *International Journal of Oral & Maxillofacial Surgery*.

[13] Goldsmith S. M., Seo B. L., Kumara De Silva R., Parachuru P., Rich A. M., Seymour G. J. (2014). Oral mycosis fungoides: report with immune profile. *Oral Surgery, Oral Medicine, Oral Pathology and Oral Radiology*.

[14] Harman M., Akdeniz S., Arslan A., Köyoğlu S. (1998). Mycosis fungoides with involvement of the oral cavity. *Journal of the European Academy of Dermatology and Venereology*.

[15] Laskaris G. C., Nicolis G. D., Capetanakis J. P. (1978). Mycosis fungoides with oral manifestations. *Oral Surgery, Oral Medicine, Oral Pathology*.

[16] Luigetti M., Cianfoni A., Scarano E. (2011). Mycosis fungoides as a cause of severe obstructive sleep apnea. *Internal Medicine*.

[17] Postorino M., Pupo L., Provenzano I. (2016). A case of oral mycosis fungoides successfully treated by combination of alemtuzumab and chemotherapy. *Annals of Hematology*.

[18] Wahie S., Lucraft H. H., Hartley C., Milne D. S., Prabhu V., Farr P. M. (2006). Oropharyngeal mycosis fungoides [5]. *Clinical and Experimental Dermatology*.

[19] Wright J. M., Balciunas B. A., Muus J. H. (1981). Mycosis fungoides with oral manifestations. Report of a case and review of the literature. *Oral Surgery, Oral Medicine, Oral Pathology*.

[20] Jawed S. I., Myskowski P. L., Horwitz S., Moskowitz A., Querfeld C. (2014). Primary cutaneous T-cell lymphoma (mycosis fungoides and Sézary syndrome): part II. Prognosis, management, and future directions. *Journal of the American Academy of Dermatology*.

